# Flash nanoprecipitation of magnetic particle imaging tracers with tunable performance

**DOI:** 10.1039/d6nr01104g

**Published:** 2026-06-24

**Authors:** Aniela Nozka, Daniela P. Valdés, Eric D. Imhoff, Andrii Melnyk, Carlos M. Rinaldi-Ramos

**Affiliations:** a Department of Chemical Engineering, University of Florida Gainesville FL 32611 USA carlos.rinaldi@ufl.edu; b J. Crayton Pruitt Department of Biomedical Engineering, University of Florida Gainesville FL 32611 USA

## Abstract

Magnetic particle imaging (MPI) is a novel biomedical imaging modality where biocompatible magnetic nanoparticle (MNP) tracers produce a quantitative signal for imaging applications like pharmacokinetics studies, blood pooling, cell tracking, and more. Improvements to tracer performance are generally made through the alteration of the synthesis mechanism and post-synthesis surface modification tailored to different applications. However, relying on synthesis alone to modify MPI performance can be difficult and time consuming. This study utilized flash nanoprecipitation, a method of MNP encapsulation, to vary the MPI performance of tracers. Herein, composite nanoparticle tracers were produced by precipitating hydrophobic iron oxide nanoparticles and homopolymer in water and stabilizing them with an amphiphilic block copolymer. By increasing the concentration of homopolymer used in precipitation, composite nanoparticle tracers with a range of physical properties and MPI performances were obtained, until a peak MPI performance was achieved and after which additional homopolymer did not further improve performance. Two pre-clinical applications of this work were demonstrated; the first was an *in vivo* imaging study and the second was color MPI. Intravenous injections show accumulation of two formulations in the liver and spleen after 24 hours, with MPI performance matching trends previously identified. Color MPI, an application of MPI whereby the signal from two MNP tracers can be separated into the signal contribution from each particle, allows for simultaneous tracking of multiple labelled species. Here, it was demonstrated using a 3D-printed mouse phantom mimicking accumulation of a two-formulation mixture in the liver, and individual particle formulations accumulating in the brain and hind flank tumor. The ratio of particles in each location was determined from the image and were comparable to ground truth values. This work demonstrates the ability of FNP to produce tunable MPI tracers, unlocking a range of applications, including pre-clinical imaging and color MPI.

## Abstract 2

Magnetic particle imaging (MPI) tracers can be formulated under different conditions *via* flash nanoprecipitation (FNP), yielding tracers with variable performance.

## Graphical abstract

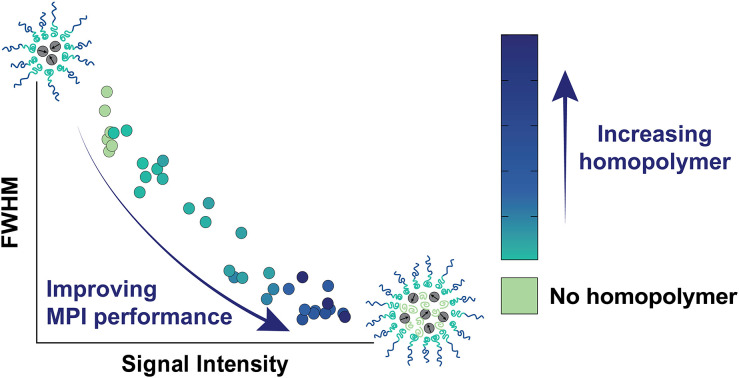

## Introduction

MPI is a novel biomedical imaging technique where magnetic nanoparticles (MNPs) respond to an alternating magnetic field to generate signal.^1^ This modality is advantageous because there is no signal attenuation due to tissue and therefore no penetration depth limitations. The signal generated in MPI is quantitative, providing information on the mass of particle tracers in each location. When overlayed with CT, the signal generated from the nanoparticles can be mapped to anatomical locations, providing information on the distribution and accumulation in the body. This technique has been used in the past to track the movement of cell therapies, image blood pooling, and map sentinel lymph nodes.^2–4^

MPI uses iron oxide nanoparticle tracers that are biocompatible and whose MPI performance does not rapidly decay as with nuclear imaging agents. These particles are currently used in various other biomedical applications as magnetic resonance imaging (MRI) contrast agents to drug carriers and agents in thermal therapies.^5,6^ Their performance in MPI is determined by the composition, size, and state of the particles. Iron oxide nanoparticles can be synthesized in a variety of methods from co-precipitation to thermal decomposition with many variations in technique.^7–12^ After synthesis, the surface can be modified depending on the application to elicit a response, target the particle to a specific tissue, increase circulation half-life, or even just make a highly hydrophobic particle into a hydrophilic package. A current limitation in the field is producing these tracers reproducibly on a scale large enough for extensive MPI experiments.

Flash nanoprecipitation (FNP) has been used to formulate nanocarriers loaded with hydrophobic components stabilized with an amphiphilic block copolymer corona, transferring stability of the constituents to aqueous solutions. FNP is advantageous compared to other methods due to its rapid formation, small size of nanocarriers produced, narrow size dispersity, and scalability. It has been studied for applications such as formulating drug carriers and RNA vaccines.^13^ FNP employs rapid micro mixing to induce high supersaturation of hydrophobic compounds in water, causing rapid aggregation of the hydrophobic components forming composite nanoparticles.^14^ The aggregation and growth of these particles is arrested by the adsorption of the amphiphilic polymer to the particle surface. For the coating of small hydrophobic molecules, FNP conditions have been widely studied to understand the effects on coating efficiency, drug delivery, and size.^14–20^ While most of the previous work on FNP focuses on the encapsulation of hydrophobic molecules, a few studies have utilized this method to encapsulate inorganic cores like MNPs to produce imaging tracers.^21–31^ A more limited number of these have used those nanoparticles as tracers for magnetic particle imaging, and of those, MPI characterization of only one formulation is mentioned.^22,23,25^ While FNP has been demonstrated for utility in formulating tracers, this process has not been systematically studied and optimized for MPI.

Advances in MPI tracers and hardware are enabling the transition of MPI from preclinical to clinical scale. Clinical scanners are in development and exploration of potential applications is ongoing.^32–35^ For many of these applications it is important to formulate MPI tracers with tuned imaging performance, tailored surface properties, and controlled size. One particularly impactful application is color MPI, wherein two particles that are distinguishable from each other can be used simultaneously and tracked as separate entities. Applications demonstrated preclinically using color MPI include imaging brain bleeds, catheters, and gastrointestinal bleeding.^4,36,37^ Color MPI has previously been demonstrated using particles produced from different syntheses which can be a laborious effort.^4,38–40^ To our knowledge, color MPI has not been demonstrated using nanoparticles formulated using the same magnetic core but different formulation conditions. The ability to modify response of tracers after they are synthesized lends to ease in manufacturing and transition to a range of pre-clinical and clinical applications.

Herein, we evaluated the role of FNP parameters in formulating composite nanoparticle tracers s. The roles of concentration of amphiphilic block copolymer (BCP) and hydrophobic homopolymer on tracer hydrodynamic size and MPI performance (sensitivity and resolution) were studied. We demonstrate scalability of these formulations by comparing the properties of tracers formulated at 1× and 5× scale. In a pilot study of *in vivo* use, mice were injected with tracers with different MPI performance, and MPI signal distribution was evaluated 24 hours after injection. Phantom studies using capillaries and 3D-printed anatomically correct mouse phantoms were used to evaluate the potential of the formulated particles for color MPI.

## Materials and methods

### Materials

Iron(iii) acetylacetonate (>98% pure) was purchased from TCI American (Portland, OR). Oleic acid (90% technical grade), sodium acetate, phosphotungstic acid hydrate, TraceCERT iron standard, 1,10-phenathroline monohydrate, and diethyl ether (certified ACS) were purchased from Sigma-Aldrich (St Louis, MO). Oleyl alcohol (80–85% technical grade), hexane (>98.5%, certified ACS), toluene (>99.5%, certified ACS), hydroxylamine hydrochloride, ethanol (200 proof), 70% Optima grade nitric acid, and tetrahydrofuran (THF, 99.8% for HPLC) were purchased from Thermo Fisher Scientific (Waltham, MA). Poly(ethylene glycol-*b*-lactic acid) (PEG-PLA, Resomer RP d 505, 16 kDa *M*_w_, 50% PEG, 1.2 PDI) and poly(lactic-*co*-glycolic) acid (PLGA, Resomer RG 502 H, PS equivalent molecular weight 13.7 kDa, 51% d,l-lactide) were purchased from Evonik (Essen, Germany). Included information is from the Certificate of Analysis for each polymer from Evonik. Ultrathin Carbon Film on Lacey Carbon Support Film (300 mesh, Gold) purchased from Ted Pella (Redding, CA). TraceCERT iron standard (Sigma-Aldrich, 56209) were purchased for use in the iron quantification assay. LS Columns were purchased from Miltenyi Biotec (Bergisch Gladbach, Germany).

### Synthesis of MNPs

The magnetic nanoparticle cores were synthesized using an esterification synthesis as previously reported.^12^ Briefly, iron oleate was prepared by combining 10.07 g of iron(iii) acetylacetonate with 40.26 g of oleic acid in a 250 mL three-neck flask and heated to 325 °C until the free oleic acid was determined to be 35% by FTIR. Then, 13 mL of oleyl alcohol were added to a 100 mL three-neck flask, placed in a heating mantle, and mixed using a magnetic stir bar and plate. The reactor's left neck was connected to a Schlenk line, and the middle neck had a rubber septum with a thermocouple. A glass stopper was added to the right neck of the reactor before starting a vacuum treatment for 1 hour while heating to 140 °C. After the vacuum treatment, the reactor was purged with Argon. Then, the reactor was wrapped in insulation before heating to the desired reaction temperature of 320 °C. After reaching the reaction temperature in the reactor, iron oleate was dripped from the middle neck of the reactor through a stainless steel needle at 0.16 mL min^−1^ using a syringe pump for a total of 50 minutes. Nanoparticle crude was washed prior to use in FNP using a solvent/anti-solvent wash procedure with magnetic separation in a circular Halbach array constructed from eight 2″ × ¼″ × ¼″ N42 neodynium magnets arranged such that each magnet is rotated 135° from the previous using a 3D-printed holder to create a quadrupole configuration. First, 1 mL of the crude from the reaction mixture was added to a 15 mL centrifuge tube with 0.3 mL of hexane and 1 mL of ethanol. The solution was vortexed before being placed in the circular Halbach array for 10 minutes. Following, the supernatant was decanted and the nanoparticles were re-suspended into 1 mL of hexane along with 1 μL of oleic acid. The solution was sonicated for 1 minute before ethanol was added and the mixture was placed back in the circular Halbach array. The addition of hexane and ethanol was repeated once more, then decanted, and the final solution was resuspended in tetrahydrofuran (THF).

### Flash nanoprecipitation of MNP nanocarriers

First, the hydrophobic constituents, MNPs (1 mg_Fe_ mL^−1^) and/or PLGA (0–20 mg mL^−1^), were dispersed in THF. Similarly, the amphiphilic block copolymer, PEG-PLA (10–65 mg mL^−1^), was dispersed in THF. One 1 mL Air-Tite syringe was loaded with the MNP/PLGA solution and another was loaded with 1 mL of the BCP solution. Two Air-Tite 1 mL syringes were loaded with 1 mL each of deionized (DI) water. A 150 mL beaker was filled with 16 mL of DI water and was placed under the mixer, acting as a quench bath for the formulation. Syringes were loaded into a multi-inlet vortex mixer (MIVM) starting with the two water syringes, placed next to each other, followed by the two THF streams, which are also placed next to each other. The syringes were simultaneously depressed at a rate of roughly 60 mL per minute and are collected in the quench bath. For the chosen flow rate, the Reynolds number is about 52 000, well above the 1600 value necessary for successful FNP.^41^

Formulation of composites in flash nanoprecipitation can produce particles without MNP cores, leading to a magnetic and non-magnetic fraction. For MPI, only the magnetic fraction is valuable and using magnetic separation on the sample removes the non-magnetic fraction. Post-formulation magnetic separation was done using LS Miltenyi columns. The LS Miltenyi column was loaded into a QuadroMacs separator and the solution was added to the column in 6 mL increments. Once the whole solution was added to the column, 6 mL of DI water was added to the column. Finally, 3 mL of DI water was added to the column and the column was removed from the QuadroMacs separator and held over the collection vial. Solution was collected until the liquid coming out turned clear again, indicating no magnetic particles were left in the column.

Formulation scale-up was tested by encapsulating 5 mg_Fe_ of MNPs with 50 mg of PLGA and 50 mg BCP. First, the 1× formulation was prepared according to the procedure above. Then, this was repeated at 5× scale where constituents were dispersed in 5 mL of THF. These solutions were loaded into 5 mL Air-Tite syringes, along with two syringes filled with 5 mL of DI water. The syringes were loaded into the MIVM and were simultaneously depressed in about 5 seconds, keeping the flow rate consistent with the 1× procedure. The mixture was collected in 80 mL of DI water. Post-formulation processing was done as described above.

### Iron quantification

The mass of iron was determined using the 1,10-phenanthroline assay. First, 10 μL of MNP or 20 μL of FNP solution were digested in 70% Optima grade HNO_3_ overnight in a heating block at 101 °C. Following digestion, 10 μL of this solution was placed in a quartz plate and liquid was evaporated in a heating block at 115 °C. The quartz plate was placed in the Opentrons OT-2 Liquid Handler (Opentrons, Long Island City, NY, USA) to complete the assay, where 46 μL of DI water was added to each sample well, followed by 30 μL of hydroxylamine solution (8.06 M). An hour after hydroxylamine addition, the liquid handler added 49 μL of sodium acetate solution (1.22 M) and 75 μL of 1,10-phenanthroline monohydrate solution (13 mM), for a total of 200 μL of liquid in each well. Simultaneously, a calibration curve was prepared where known iron stock (TraceCERT) samples were added to wells and underwent the same procedure in the robot. The plate was removed from the robot and placed in a Spectramax-M5 Plate Reader (Molecular Devices, San Jose, CA, USA), and the end-point absorbance was measured at 508 nm. All samples and standards were prepared in triplicate to serve as technical replicates.

### Dynamic light scattering (DLS)

Hydrodynamic diameter was recorded using a DynaPro Plate Reader III (Wyatt Technology, Goleta, CA, USA). About 15 μL of MCNC solution is loaded into a well in a clear bottom 96-well plate with 85 μL of deionized water. Once all samples were loaded, the plate was placed in the plate reader and hydrodynamic diameters were measured *via* the dynamic light scattering. The intensity weighted hydrodynamic diameters are reported.

### Transmission electron microscopy (TEM)

MNPs were imaged using TEM prior to being used in FNP. Here, 5 μL of washed MNP solution was dropped onto a 200-mesh carbon-coated copper grid. Images were analyzed using MATLAB code to determine the diameter of over 500 particles to generate a histogram of the sample's size. Samples were imaged on a FEI Talos F200i S/TEM at 200 kV and up to 120k× magnification.

Select particle formulations made from FNP were imaged using TEM to assess composite nanoparticle morphology. To prepare the grids, 5 μL of composite nanoparticle solution was loaded into a pipette and placed onto a 300-mesh ultrathin carbon film on lacey carbon support gold grid, left for 1 minute, wicked away using the edge of a Kimwipe, and allowed to dry completely. Negative staining was similarly done by placing 5 μL of a 2% phosphotungstic acid solution adjusted to pH 6 onto the same grid, waiting for 30 seconds, wicking away, and allowing to dry completely. Images were collected to qualitatively understand composite nanoparticle composition.

### Magnetic particle imaging relaxometry

To screen particles performance in magnetic particle imaging, all samples were scanned in a Magnetic Insight Momentum X-space scanner (Magnetic Insight, Alameda, CA, USA) using the MPI RELAX mode to determine the signal intensity and full-width-a-half-maximum (FWHM) for each particle, where the FWHM corresponds to the resolution of the tracer. First, particle solutions were added to a microcentrifuge tube at volumes between 10 and 200 μL One at a time, the centrifuge tubes are loaded into the MPI and the RELAX scan is run. The output of this scan is a point-spread function (PSF), where the peak of the curve corresponds to the signal intensity, which was normalized to iron mass.

### Magnetic particle imaging 2D scans

To generate images of select MPI tracers, the 2D scan feature of the momentum was used. First, 5 μL of particle solutions prepared to the same concentration were loaded into a 3 cm long capillary tube. The capillary was loaded vertical in a 3D printed stand within a custom MPI bed, and the scans were run. For resolution phantom images, the capillaries were laid flat on a 3D-printed insert with spacers to separate two capillaries at known distances. The distances recorded are from the center of one capillary to the center of the next. Images were collected at close distances and capillaries were moved farther apart and scanned until the measured signal between the two capillaries was less than 50% of the maximum signal.

### *In vivo* imaging

Two C57BL/6 wild type mice, obtained from Jackson Laboratory, were injected intravenously with composite nanoparticle tracer formulations prepared in 200 μL of saline, containing 100 μg_Fe_. Mice were anesthetized with isoflurane, placed in a 3D-printed MPI bed, and imaged in the MPI followed immediately, without waking, by a CT scan (IVIS® Spectrum CT, PerkinElmer, Waltham, MA, USA). After *in vivo* imaging, mice were euthanized with isoflurane overdose. All animal procedures were performed in accordance with the Guidelines for Care and Use of Laboratory Animals of the University of Florida Institutional Animal Care and Use Committee and approved by the Animal Ethics Committee of the University of Florida.

### Color MPI

Color MPI relied on unmixing the signal generated by two different tracers that are colocalized in the same volume or image. For the first experiment, two particle formulations were chosen, the 10 mg mL^−1^ PLGA formulation (labeled “formulation A”) and the 0 mg mL^−1^ PLGA formulation (“formulation B”). Capillaries with a 10 μL volume of 100, 75, 50, 25 and 0 wt% mixtures for one formulation and the complementary amount for the other were prepared for this study. They were scanned in the Momentum at excitation field amplitudes from 10–16 mT and a gradient of 5.7 T m^−1^.

We also tested the color MPI capabilities in a phantom mimicking mouse anatomy, for which we used a 3D-printed anatomically correct mouse phantom based off one that has been previously reported.^42^ First, capillary tubes were prepared with a 100 wt% in formulation A or formulation B at a total mass of 5 μg. The capillary with formulation A was loaded into the brain cavity and the capillary with formulation B was placed in the flank tumor area. The liver cavity was filled with a 50 wt%/50 wt% mixture of formulations A and B. A 2D High Resolution MPI and CT scan was taken. Analogous scans were taken for reference capillary and liver samples with 100 wt% formulation A or B and the same total mass as in the experiment.

The maximum signal for each excitation field amplitude *H*_i_ and formulation A mass ratios *m̄*_*j*_, *s*_max_(*H*_i_, *m̄*_*j*_), was determined, where *i* = 0, 1, …, *M* and *j* = 0, 1, …, *N*. The 100 wt% sample data, *s*_max_(*H*_i_, 1), was used to obtain the characteristic signal vector for formulation A at the different excitation amplitudes, 
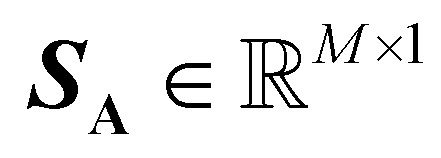
 Analogously, the 0 wt% sample data was used to get the characteristic signal vector for formulation B, 
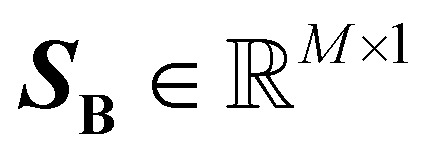
. Explicitly, ***S***_A_ = [*s*_max_(*H*_0_,1), *s*_max_(*H*_1_,1), …, *s*_max_(*H*_*M*_,1)]^*T*^ and ***S***_B_ = [*s*_max_(*H*_0_,0), *s*_max_(*H*_1_,0),…, *s*_max_(*H*_*M*_,0)]^*T*^.

The maximum signal per unit total mass for mixture *i*, 
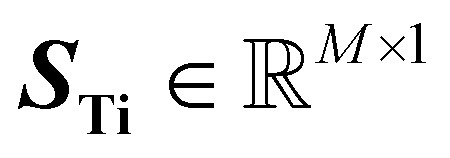
, can be written as the sum of the signal contributions from each particle in the mixture, that we can obtain from multiplying the reference signal by the corresponding mass,

1 ***S***_**Ti**_ = ***S***_**A**_*m̄*_*i*_ + ***S***_**B**_(1 − *m̄*_*i*_).

Considering eqn (1) for each of the *N* formulation A mass ratios, we can write the system of equations,

2 ***S***_**TB**_ = ***S***_**AB**_***m̄***,

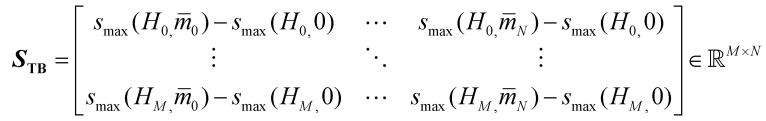

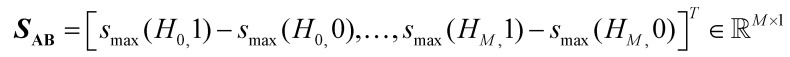
 and 
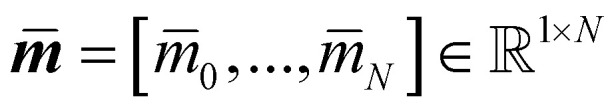
.

Eqn (2) has a non-trivial solution when the rank of ***S***_TB_ is equal to 1 and ***S***_AB_ is not null. In that case, the mass ratios can be obtained as,3
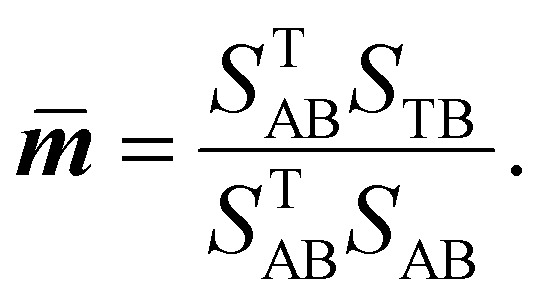


## Results and discussion

### Size of composite nanoparticles formulated *via* FNP depends on homopolymer concentration

**Fig. 1:**
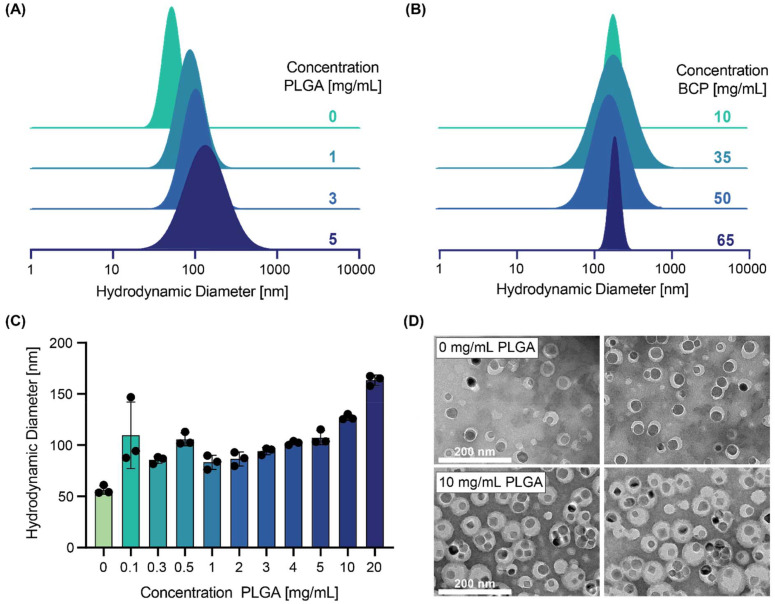
Size of composite nanoparticles varies as a function of the concentration of homopolymer. Representative normalized hydrodynamic diameters (intensity weighted average) of composite nanoparticles formulated with different (A) PLGA concentrations and (B) block copolymer concentrations. (C) Hydrodynamic diameters of all samples formulated across increasing concentrations of PLGA. (D) Representative transmission electron microscopy images of a sample formulated with 0 mg mL^−1^ PLGA and a sample formulated with 10 mg mL^−1^ of PLGA.

Magnetic nanoparticles were synthesized *via* an esterification method described by Velazquez-Albino *et al.*^12^ MNPs were cleaned using solvent/anti-solvent washes to remove extra organics from synthesis and transfer into tetrahydrofuran for use in FNP. Composite nanoparticles were formulated *via* FNP using the multi-inlet vortex mixer, where four inlet streams were rapidly mixed against each other and the product was collected in a quench volume (Scheme S1). The multi-inlet vortex mixer allows for modularity, as opposing streams do not need to match in momentum. Two streams contained hydrophobic constituents suspended in THF and the other two streams contained deionized water. The hydrophobic constituents used in these formulations were PLGA and MNPs in one THF stream, and the amphiphilic stabilizing copolymer, PEG-PLA, in the other THF stream. When these streams are rapidly mixed against the water streams, the hydrophobic constituents aggregate and the amphiphilic polymer stabilizes the surface, inhibiting further growth and rendering them compatible with water. After formation in FNP, the sample is purified using magnetic separation, filtering out excess polymer that is not encapsulated and any MNP free particles. Samples then underwent physical and magnetic characterization. We chose to test the effect of varying PLGA and PEG-PLA concentrations on the physical and MPI properties of magnetic composite nanoparticles. For this, homopolymer was added at concentrations of 0–20 mg mL^−1^ and concentrations of PEG-PLA used were between 10–65 mg mL^−1^ (parameters shown Table S1). To test the effect of homopolymer concentration on the properties of the composite nanoparticles, the homopolymer was varied and the BCP was kept at 10 mg mL^−1^, and to test the effects of BCP on properties, the homopolymer was kept at 20 mg mL^−1^. As seen in Fig. 1A–C, adding more homopolymer to the formulation increased the hydrodynamic diameter of the composite nanoparticles, whereas increasing the concentration of BCP in the formulation had little effect on the size of the composite nanoparticles. As the concentration of homopolymer in a formulation is increased while keeping other parameters constant, there is more core material that can be in each particle. More core material in each particle would lead to an increase in the size of the particles, which agrees with trends seen here. Importantly, all particle hydrodynamic diameters remain below 200 nm, critical for use in biological applications where sterilization often relies on passing material through a 0.22 µm filter. Fig. 1D shows representative TEM images of composite nanoparticles formulated with 0 mg mL^−1^ and 10 mg mL^−1^ of PLGA, showing encapsulated nanoparticles. The MNPs have a physical diameters of approximately 19 nm (Fig. S1). This study has several limitations that could guide future studies. Here, the MNPs used were made using esterification and are on the size of 18–20 nm. Esterification synthesis of iron oxide nanoparticles produces particles coated with oleic acid, like a more common synthesis technique, thermal decomposition synthesis, so we would expect that these should behave similarly for similar sized particles. Oleic acid makes the MNPs a highly hydrophobic component which is amenable to formulation using FNP. The effect of size on the ability to encapsulate MNPs will require further experimentation as this has been suggested to be a factor in the ability to perform flash nanoprecipitation of larger inorganic particles.^29^ However, our initial testing with particles formulated *via* esterification demonstrates relationships between homopolymer addition and the size of the nanocomposite, consistent with previous reports on the effect formulation parameters on size.^17^

### Homopolymer concentration tunes MPI performance of magnetic composite nanoparticles

**Fig. 2:**
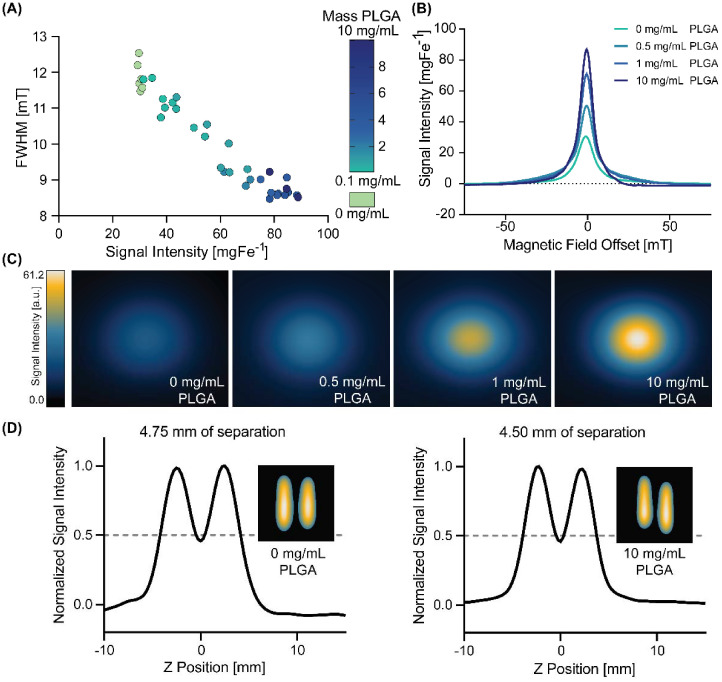
MPI performance improves with increasing PLGA concentration. (A) The MPI performance (signal intensity and FWHM) shown as homopolymer concentration varies, where samples in the bottom right quandrant have better MPI performance. (B) Representative point-spread functions of samples prepared with increasing PLGA concentration. (C) 2D MPI images of samples prepared with increasing concentrations of PLGA. (D) Resolution phantom images show separation of signal at different distances of separation for the 0 mg mL^−1^ PLGA and 10 mg mL^−1^ PLGA tracers.

All composite nanoparticles were screened for MPI performance using MPI RELAX mode to obtain a point-spread function (PSF) for each sample, from which the signal intensity and full-width-at-half-maximum (FWHM) were extracted to draw conclusions about MPI performance. All RELAX scans were normalized to iron mass to facilitate comparisons between samples. Fig. 2A shows the signal intensities and FWHM of all the samples at constant BCP and varying homopolymer concentrations. This shows the MPI performance improving (increased signal and decreased FWHM) as the concentration of homopolymer is increased from 0–10 mg mL^−1^. The signal intensity improves from 30 mg_Fe_^−1^ to almost 90 mg_Fe_^−1^ and the FWHM decreases from nearly 12 mT to 8.5 mT. Not only is there a large improvement from 0 mg mL^−1^ PLGA to 10 mg mL^−1^ PLGA, but there is control over the performance between these two extremes by adding variable amounts of homopolymer. Between samples, we see significant differences between signal intensity, indicating the samples are statistically different (Fig. S2). Composite nanoparticles made with 3, 4, 5, and 10 mg mL^−1^ of homopolymer had similar performance, indicating there is a limit of performance for these particles of around 85 mg_Fe_^−1^ for signal intensity and around 8.5 mT for FWHM (Fig. S3-S4). Increasing the concentration of BCP used in the formulation at constant homopolymer and MNP masses had no effect on MPI performance (Fig. S5). This suggests BCP does not play a significant role in tuning MPI performance. Samples were prepared in triplicate to evaluate formulation reproducibility, and all coefficients of variance were below 15% for signal intensity and FWHM (Table S3). From these RELAX scans, a few representative PSFs are shown in Fig. 2B to further demonstrate tunable MPI performance among formulations. Select samples underwent 2D MPI imaging to generate an image of each tracer and confirm their performance, as shown in Fig. 2C. Samples were prepared in microcentrifuge tubes at the same total iron mass and solution volume. A consistent color scale was applied, where the minimum/maximum is the lowest/highest signal across the four images. The 10 mg mL^−1^ PLGA sample is brightest and the 0 mg mL^−1^ formulation is dim in comparison, demonstrating control over signal intensity across the samples. Our data suggests that the increasing of homopolymer changes MPI performance and we hypothesize this is due to changing the interparticle interactions of the MNP in the core of the composite nanoparticles.

To demonstrate the control over resolution in these tracers, the best (10 mg mL^−1^ PLGA) and worst (0 mg mL^−1^ PLGA) tracers, in terms of intensity and resolution, were compared (Fig. 2D). The 10 mg mL^−1^ PLGA formulation was loaded into two capillary tubes, and images were taken in the MPI at known distances, until the signal between the two capillaries was distinguishable, taken to be when the signal between the two tubes dipped below 50% of the max signal. All distances reported are the center-to-center distance between capillaries. Neither formulation had distinguishable signal at 4.25 mm of separation (Fig. S6). However, at 4.50 mm of separation the 10 mg mL^−1^ PLGA sample is distinguishable, with the signal between the two capillaries dipping below 50% of the maximum signal. The 0 mg mL^−1^ PLGA sample does not have separation of signal until they are separated by 4.75 mm.

This work demonstrates the use of FNP to produce tracers with differing MPI performance. While the idea of using FNP to encapsulate and stabilize hydrophobic species, such as MNPs, is not new, the role of formulation conditions on MPI performance and the tradeoff between size and performance shown here has not been reported previously. Varying formulation parameters in FNP is simple; it does not require significant changes to process setup or introducing new materials, which has advantages for manufacturing under regulatory frameworks. By solely increasing the concentration of homopolymer, we demonstrated the ability to reproducibly control tracer performance and identify an optimal performance. This approach is unique in its ability to use the same iron oxide core to formulate a series of MPI tracers with distinct performance from the same basic components (the MNP, a hydrophobic homopolymer and an amphiphilic block copolymer).

### MPI performance is preserved upon scale-up

**Fig. 3:**
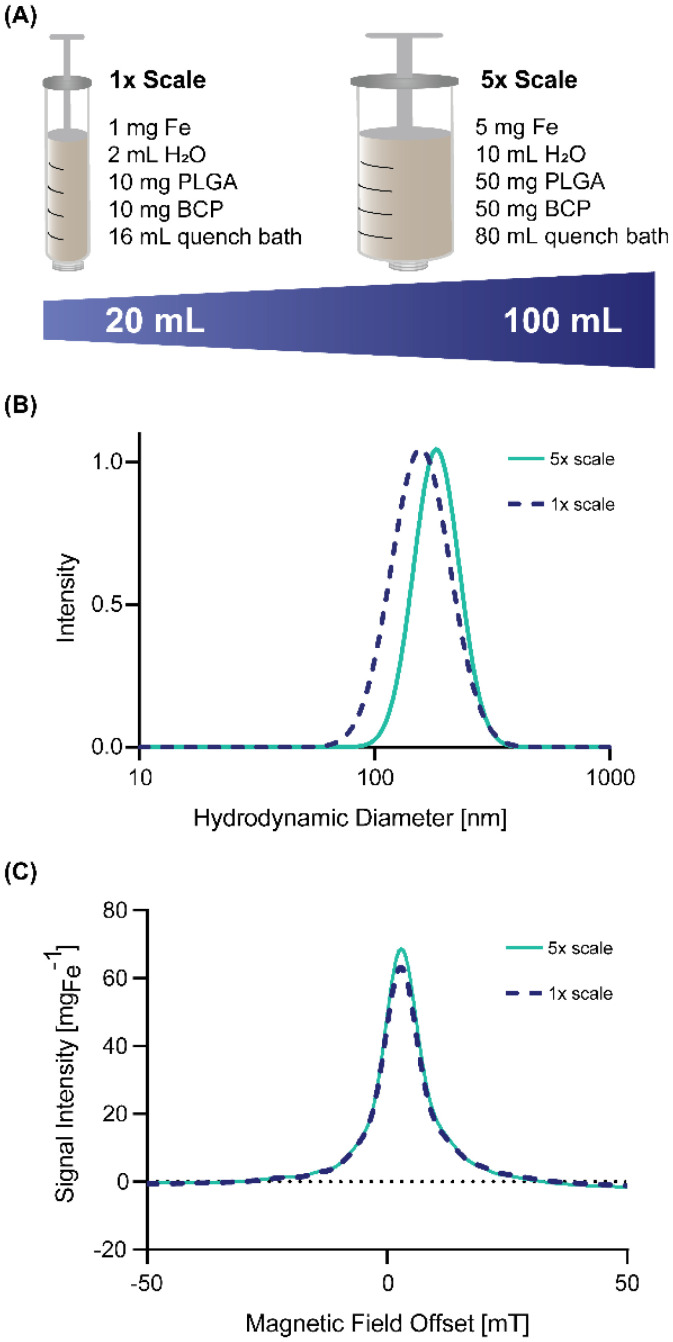
Scale-up of flash nanoprecipitation produces composite nanoparticles of similar size and performance. (A) Representation of scale-up and conditions used in the scale up of FNP from 1× to 5×, where on the 1× scale there is 20 mL of formulation to process. Scaling up to 5× increases this to 100 mL of liquid to process after formulation. (B) Hydrodynamic diameter normalized histograms for the 1× and 5× formulations. (C) MPI point-spread functions for 1× and 5× formulations.

One of the advantages of FNP is that formulations can be tested on a small scale and later scaled up reproducibly. To demonstrate scalability for MPI tracer formulation using flash nanoprecipitation, we scaled up the procedure by 5×, using 5 mL syringes. All constituents and volumes used in the procedure were scaled by 5×, as seen in Fig. 3A. The syringes were depressed at the same flow rate as previously used. The collected sample was filtered using the same magnetic separation columns as were used for the 1× formulations. A 1× batch was prepared under the same conditions to compare all results. The hydrodynamic diameters were 160 nm and 190 nm for the 1× and 5× batches respectively, as seen in Fig. 3B. Fig. 3C shows the PSFs, where the 1× and 5× formulations have 63.3 mg_Fe_^−1^ and 68.6 mg_Fe_^−1^ signal intensity respectively. When producing particles at large scale and with syringe pumps, it is good practice to discard the start-up volume, which was not done in the 1× or 5× formulations. This may contribute to the slight differences in size for these samples and should be considered in the design of future experiments. Challenges in scaling up flash nanoprecipitation include post-formulation processing of the large volumes produced. To further scale-up this reaction, syringe pumps can be used which allow for larger sized syringes to be used by increasing the run-time of the formation. Additionally, mixer geometry can be scaled to enable throughput of larger volumes. While our scale-up did not necessitate the use of syringe pump or changing mixer geometry, incorporating and validating these methods is necessary for further increases in production. We demonstrate that FNP is amenable to direct scale-up by increasing processed volume without changing formulation conditions, enabling identification of lead tracer formulations followed by production at quantities required for further testing, adding to further advantages of using this method.

### *In vivo* signal intensity varies with formulation conditions

**Fig. 4:**
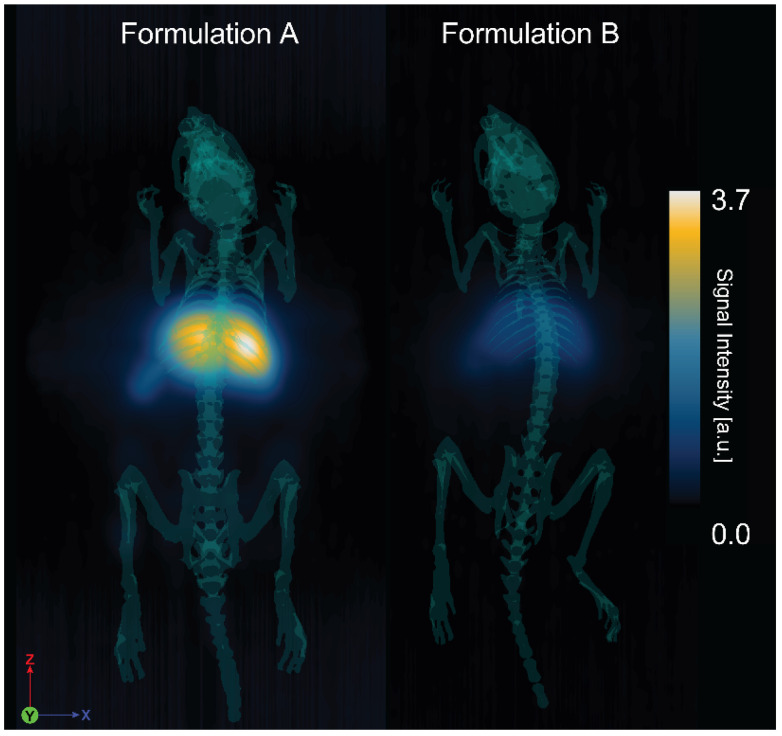
*In vivo* imaging shows accumulation of particles from different formulations in liver and spleen. MPI 3D images overlayed with skeleton segments from CT image for anatomical referencing shows accumulation of the particles in the liver and spleen 24 hours after injection. For particles injected at the same dose, the difference in signal intensity is due to the performance of the formulation used.

The best and worst performing nanoparticle formulations, formulation A (10 mg mL^−1^ PLGA) and formulation B (0 mg mL^−1^ PLGA), were administered (200 μL, 100 μg_Fe_) *via* tail vein injection in a pilot study. These formulations were chosen due to their vast difference in MPI performance. Twenty-four hours following injection, mice were placed into the MPI for a 3D MPI scan, followed immediately by a computed tomography (CT) scan. Images for each mouse were loaded into 3D Slicer, a biomedical image rendering and segmentation software, where the CT and MPI images were overlayed for each mouse using a registration matrix obtained for an independent set of fiducials and Slicer's Landmark Registration tool. The MPI signal was thresholded to show signal above 50% of the maximum, and the color lookup tables for the images were adjusted to be in the same range. In Fig. 4, there is a dim signal in the mouse receiving formulation B and a bright signal in the mouse receiving formulation A. Both formulations show terminal accumulation in the liver and spleen as expected for particles of this size. The trends in performance agree with the performance identified in the RELAX scans for each of these formulations and demonstrate that these formulations exhibit similar accumulation patterns *in vivo*. While the total signal is greater for formulation A, both produce measurable signal *in vivo*, when color lookup tables are adjusted for their corresponding dynamic ranges (see Fig. S7). We demonstrate a proof-of-concept *in vivo* imaging of particles with varying MPI performance, however, further testing across formulations could be done to further improve this concept.

### Composite nanoparticles formulated with FNP can be used as tracers for color MPI

**Fig. 5:**
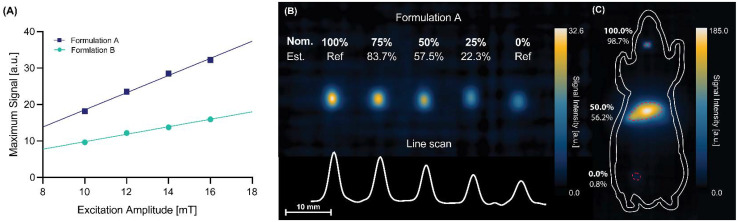
Signals from two different MPI tracers can be distinguished and used for color MPI. (A) Maximum signal intensity for each sample vs the excitation amplitude the image was taken at, fitted with a linear regression. The best fit lines have an *R*^2^ value of 0.9957 for formulation A (10 mg mL^−1^ PLGA) and 0.9934 for formulation B (0 mg mL^−1^ PLGA). (B) 2D MPI image of all mixtures taken at an excitation amplitude of 16 mT, and the subsequent line scan for the image, demonstrating how increasing amounts of formulation A in the mixture increases the signal intensity. Nominal mass ratio (Nom.) and estimated mass ratio calculated from color MPI image analysis (Est.) are shown. (C) A 2D MPI image of a mouse phantom with capillaries in the brain and tumor, and a cavity filled with a 50%/50% mixture of formulation A and formulation B. The brain capillary contains 100% formulation A and the tumor capillary contains 100% formulation B. The nominal (bold) and estimated mass ratios are shown next to the signal for each location.

Select particle formulations were screened for use as color MPI tracers. Magnetic composite nanoparticles were screened by taking 2D MPI scans at a range of excitation amplitudes and fitting the results of the maximum signal to a linear regression. From this, the 10 mg mL^−1^ (formulation A) and 0 mg mL^−1^ formulations (formulation B) were chosen as candidates for color MPI as their responses varied most of all the samples (Fig. 5A). Five different mixtures were prepared from formulations A and B, at 100%, 75%, 50%, 25% and 0% of Formulation A in the mixture by adding each particle at the appropriate ratio. The samples were then scanned at excitation amplitudes between 10–16 mT and the peak signal for each sample was determined. We obtained the characteristic signal vector from the neat samples at the different excitation field amplitudes and used these vectors and the peak values for the mixtures to solve the system of equations for the mass ratios as described under Materials and Methods. An example of a 2D MPI image of the mixtures is shown in Fig. 5B, taken at an excitation amplitude of 16 mT. The signal decreased as the mass of the 0 mg mL^−1^ PLGA tracer increased, in agreement with previous MPI scans where this formulation had a lower specific signal. The line scan for this image shows that the peak signal from each sample is distinguishable, as the signal intensity dips to baseline between each peak.

For the 75% mixture of the 10 mg mL^−1^ PLGA tracer and 25% of the 0 mg mL^−1^ PLGA tracer, the percentages calculated from MPI signal unmixing were 83.7%/16.3%. For the 50%/50% mixture, the percentages calculated were 57.5%/42.5%. The last mixture was calculated to be 22.3%/77.7%. These results are close to the ground truth values, demonstrating the ability to accurately distinguish the signal generated from two particles in one image. These results demonstrate that the performance of tracers formulated with the same iron oxide cores can be modified through simple post-synthesis FNP, enabling color MPI, which has previously only been demonstrated using different iron oxide cores.

Next, a 3D-printed mouse phantom with cavities in locations representing the brain, liver, and a hind-flank tumor was used to demonstrate a potential application of color MPI, shown in Fig. 5C. Here, the two particle formulations described previously were used and were prepared as follows. A capillary tube containing 100% of the 10 mg mL^−1^ PLGA particle sample was placed in the brain; a capillary tube with 100% of the 0 mg mL^−1^ PLGA particle formulation was placed in the hind flank tumor. A 50%/50% mixture was prepared of the two particles and was used to fill the liver cavity. This was done to mimic a situation where one particle targets the brain, the other particle accumulates in the tumor, and they both are accumulated and removed by the liver. We applied the same method as previously described to determine the percentage of each particle in the brain, tumor, and liver. The ratio in the liver was calculated to be 56.2% of the 10 mg mL^−1^ PLGA particle (ground truth 50%). The brain was found to be 98.7% of the 10 mg mL^−1^ PLGA particle (ground truth 100%), and the tumor was found to be 0.8% of the 10 mg mL^−1^ PLGA particle (ground truth 0%). This demonstrates the utility of color MPI analysis in determining how much of two different particles accumulate in different spots throughout the mouse anatomy.

Formulation of MPI tracers with tunable performance enables applications where multiple particles are simultaneously tracked through color MPI. For example, one could track two distinct cell types, as in some adoptive cell therapies that combine activated T cells and dendritic cell vaccines,^43^ in tracking two drug carriers with different *in vivo* target organs, such as one particle targeting lymphoid organs for immunotherapy and another targeting cancer tumors for cytotoxic effect, or competitive accumulation in the same tissue between two carriers. Here we demonstrated color MPI using two formulations corresponding to extremes of MPI performance. However, as algorithms and methods for color MPI improve, one can envision formulation of multiple distinguishable tracers that would unlock even more applications.

## Conclusions

Here, we reported the use of flash nanoprecipitation to produce composite nanoparticles for MPI with tunable performance, controlled *via* the amount of homopolymer added to the formulation. Increasing homopolymer concentration while keeping all other formulation parameters the same increases the hydrodynamic diameter of the composite nanoparticles and improves the MPI performance. The scalability of this technique for the application of producing composite nanoparticles was also demonstrated, allowing conditions to be tested on a small scale before identifying optimal conditions which can later be scaled to reach experimental level production. We demonstrate that the difference in MPI performance between two formulations remains *in vivo*, while having similar terminal accumulation in the liver and spleen. Using FNP to produce MPI composite nanoparticles with varying response of signal intensity with excitation amplitude lends to the ability to unmix signals generated from a mixture of particles and subsequently calculate the mass of each particle in the mixture. We demonstrate this in a 3D-printed mouse phantom for the application of accumulation of two different particles in brain, liver, and tumor. These results demonstrate the application of flash nanoprecipitation in the formulation of MPI tracers for biomedical applications.

## Author contributions

A. N. and C. M. R. R. conceptualized and designed the experiments. E. D. I. synthesized the magnetic nanoparticles and collected TEM images. A. N. performed flash nanoprecipitation to create the formulations. A. N. performed MPI RELAX and 2D scans. A. N. and A. M. performed resolution phantom scans. A. N. performed *in vivo* experiments. A. N. performed color MPI experiments. A. N. performed scalability tests. A. N. analyzed MPI RELAX, 2D, and 3D images and *in vivo* images. E. D. I. analyzed TEM images. D. P. V. analyzed color MPI images. A. N. analyzed dynamic light scattering data. A. N. and C. M. R. R. contributed equally to the visualization of the data. A. N. wrote the original draft, with main review and editing from C. M. R. R, and review from all co-authors. The manuscript was written through contributions of all authors. All authors have given approval to the final version of the manuscript.

## Conflicts of interest

CRR is an inventor on patents (US 10,634,742 B2, US 10,765,744 B2, US 11,305,351 B2, US 11,311,630 B2, US 12,072,400 B2) and patent applications (US 2022/0287969 A1, US 2023/0355811 A1), which are awarded or submitted in whole or in part to the University of Florida and are related to magnetic nanoparticles or magnetic particle imaging. The university may benefit financially from their commercialization, and the author could benefit under the university’s patent policy. All other authors declare that they have no other competing interests.

## Supplementary Material

NR-018-D6NR01104G-s001

## Data Availability

Data for this article are available at Dryad at https://doi.org/10.5061/dryad.573n5tbqp. 3D printed bed models used for MPI and CT scans can be found in a GitHub repository https://github.com/RRL-UF/RRL-Momentum-Beds/. Supplementary information (SI): flash nanoprecipitation formulation scheme, formulation conditions table, MNP size distribution, statistical analysis of MPI signal between formulations, formulation MPI property plots, coefficient of variance within formulations, renders of *in vivo* images. See DOI: https://doi.org/10.1039/d6nr01104g.
