# Supplementary material for: Flash nanoprecipitation of magnetic particle imaging tracers with tunable performance
Source: Nanoscale. 2026 Jun 24;18(30):16157–67. doi: 10.1039/d6nr01104g (PMC13339030; doi:10.1039/d6nr01104g)
Supplement: NR-018-D6NR01104G-s001 [file NR-018-D6NR01104G-s001.pdf]

This journal is © The Royal Society of Chemistry 2026

Flash nanoprecipitation of magnetic particle imaging tracers with tunable performance

Aniela Nozka[1], Daniela P. Valdes[1], Eric D. Imhoff[1], Andrii Melnyk[1], and Carlos M. Rinaldi-Ramos[1,2,*]

[1]Department of Chemical Engineering, University of Florida, Gainesville, FL 32611, USA

[2]J. Crayton Pruitt Department of Biomedical Engineering, University of Florida, Gainesville, FL 32611, USA

Supplementary Information

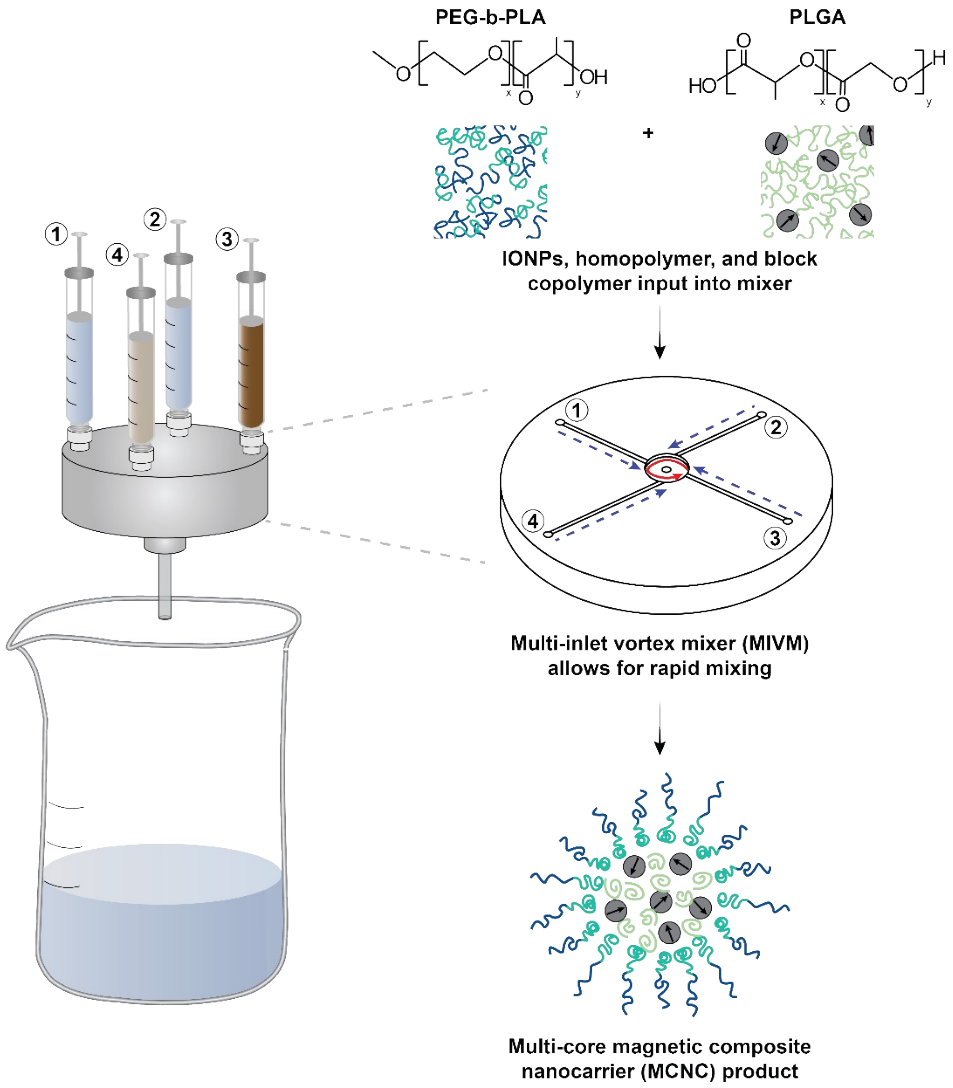

Schematic S1. A depiction of the flash nanoprecipitation procedure using an MIVM where two streams are water and two contain the THF solvent with the MNPs and homopolymer or the amphiphilic block copolymer. Polymer structures are shown on the right along with a diagram depicting the mixing within the MIVM, and finally below a depiction of the nanocluster product.

Table S1. Formulation parameters used in the experiments, all formulated with 1 mg of iron in 1 mL volume per syringe.

Mass PLGA [mg] \ Mass Block Copolymer [mg]	10	35	50	65
0	x			
0.1	x			
0.3	x			
0.5	x			
1	x			
2	x			
3	x			
4	x			
5	x			
10	x			
20	x	x	x	x

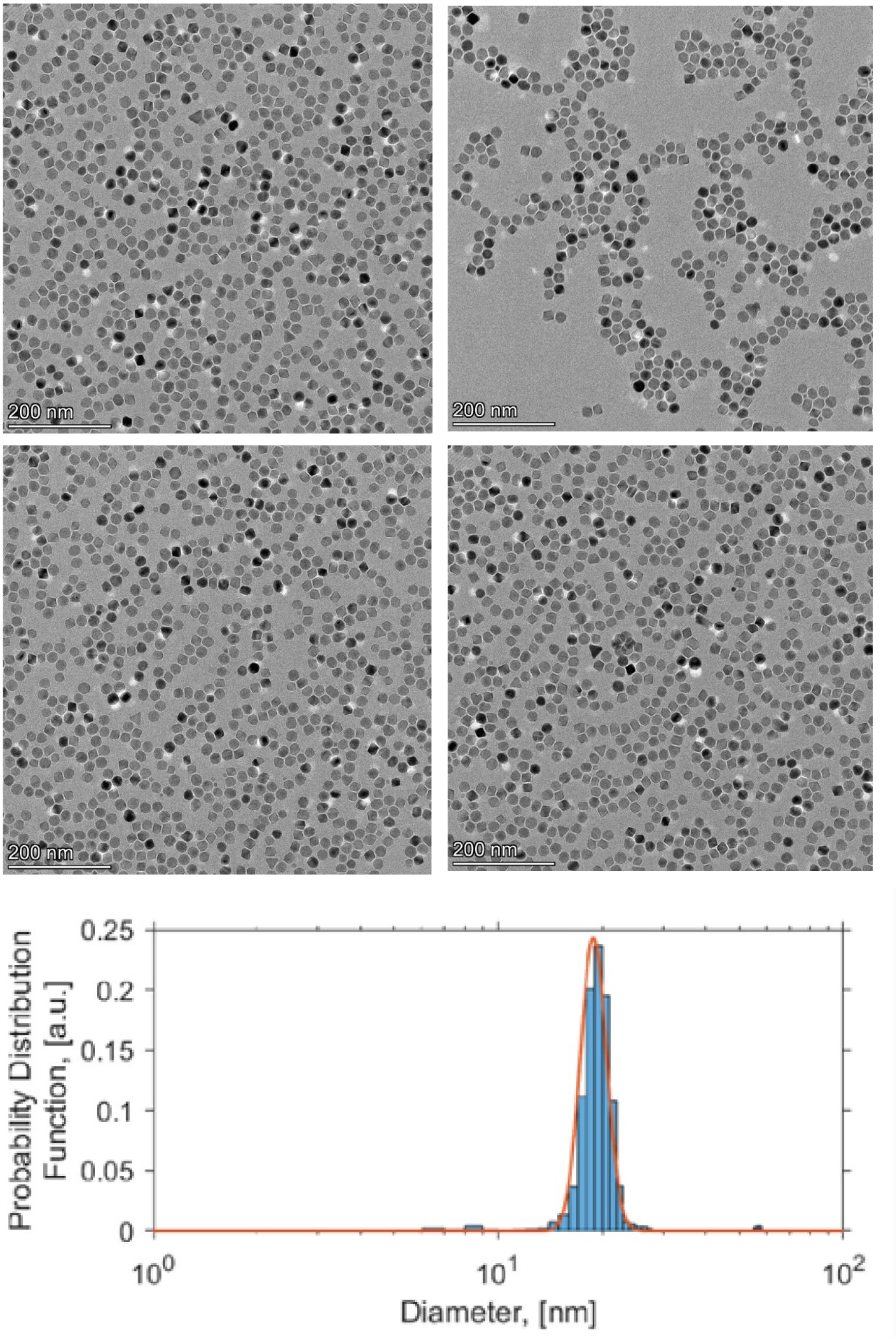

Figure S1. TEM analysis of particles shows MNP physical diameter of 19 nm.

Table S2. Statistically significant differences in MPI signal intensity are seen between formulations with different compositions of homopolymer added, while keeping iron oxide core concentrations consistent. Statistical analysis done using one-way ANOVA with a normal distribution assumption on GraphPad Prism.

Tukey's multiple comparisons test	Mean diff.	95.00% CI of diff.	Below threshold?	Summary	Adjusted P Value
0 mg/mL PLGA vs. 0.5 mg/mL PLGA	-4.624	-15.30 to 6.051	No	ns	0.5399
0 mg/mL PLGA vs. 1 mg/mL PLGA	-34.25	-44.92 to -23.57	Yes	****	<0.0001
0 mg/mL PLGA vs. 10 mg/mL PLGA	-53.64	-64.32 to -42.97	Yes	****	<0.0001
0.5 mg/mL PLGA vs. 1 mg/mL PLGA	-29.63	-40.30 to -18.95	Yes	****	<0.0001
0.5 mg/mL PLGA vs. 10 mg/mL PLGA	-49.02	-59.69 to -38.34	Yes	****	<0.0001
1 mg/mL PLGA vs. 10 mg/mL PLGA	-19.39	-30.07 to -8.719	Yes	**	0.0018

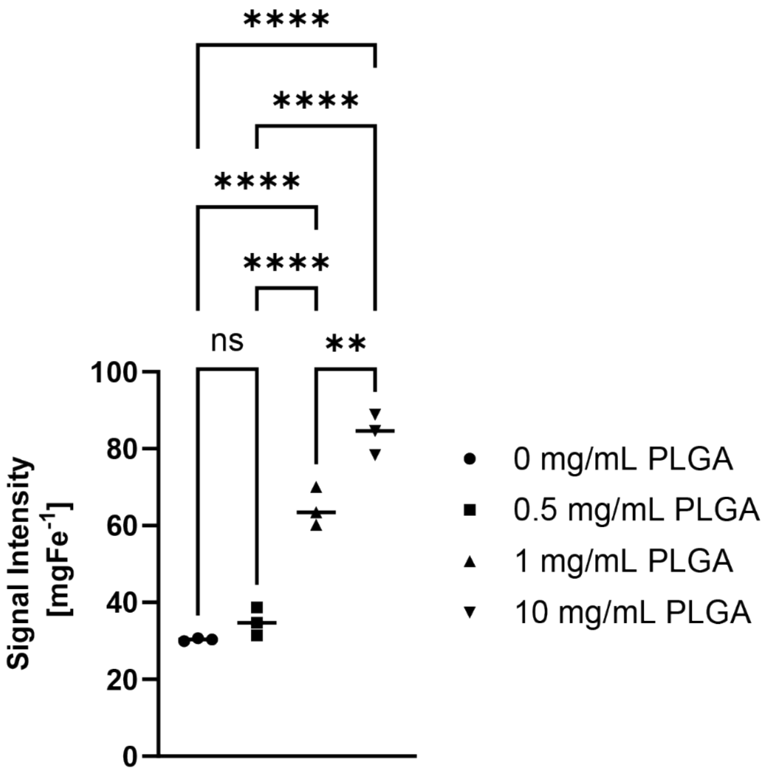

Figure S2. Statistically significant differences in MPI signal intensity are seen between formulations with different compositions of homopolymer added, while keeping iron oxide core concentrations consistent. Statistical analysis done using one-way ANOVA with a normal distribution assumption on GraphPad Prism.

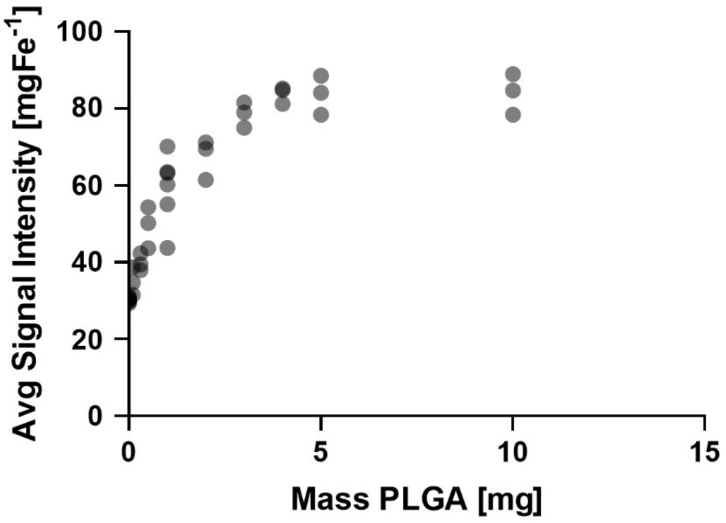

Figure S3. Signal intensity of particles formulated with different concentrations of homopolymer for all samples, showing a plateau in signal intensity around 85 $mgFe^{-1}$.

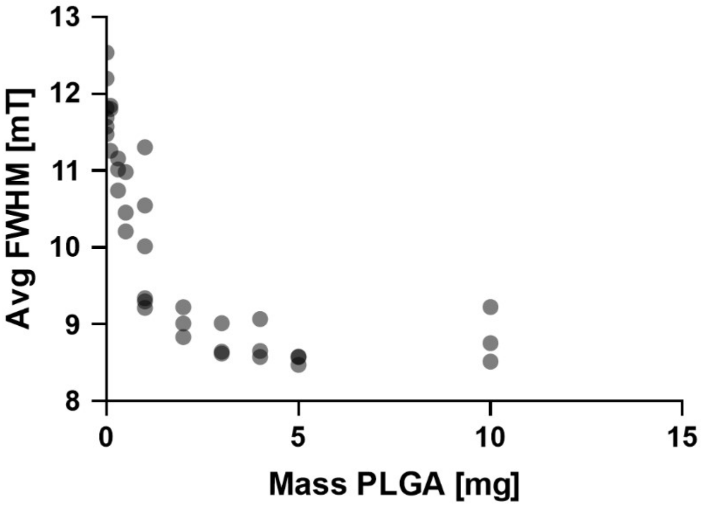

Figure S4. Full width at half maximum in mT of particles formulated with different concentrations of homopolymer for all samples shows a plateau of FWHM around 8.5 mT.

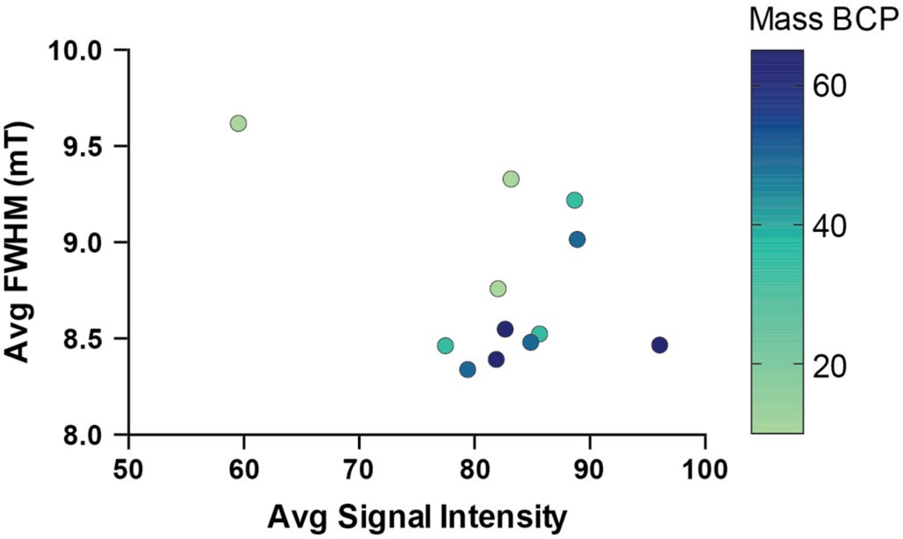

Figure S5. The MPI performance of particles formulated with different concentrations of block copolymer for all samples with 20 mg/mL of PLGA homopolymer and 1 mg/mL of iron show no apparent trend.

Table S3. Coefficient of variance calculated for each level of PLGA using the average signal intensity and FWHM for each formulation.

	Coefficient of Variance [%]	
**Mass of PLGA [mg/mL]**	**Signal Intensity**	**FWHM**
0	1.20	1.47
0.1	10.33	2.81
0.3	5.54	1.93
0.5	10.91	3.74
1	7.79	0.69
2	7.73	2.16
3	4.22	2.53
4	2.63	3.02
5	6.07	0.71
10	6.32	4.09

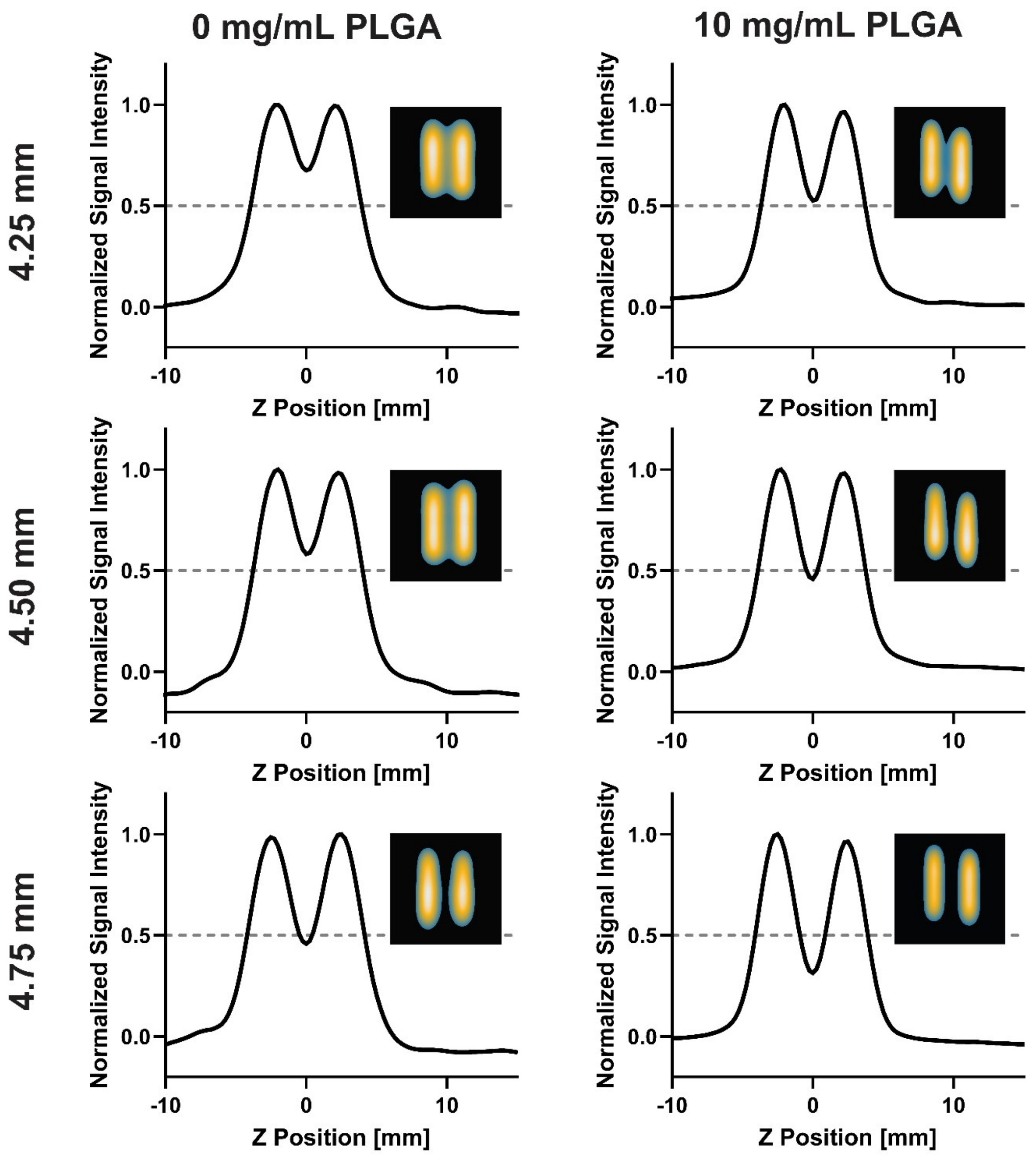

Figure S6. MPI scans of capillaries separated at known center-to-center distances, filled with 5 uL of tracer to determine the resolution of each formulation.

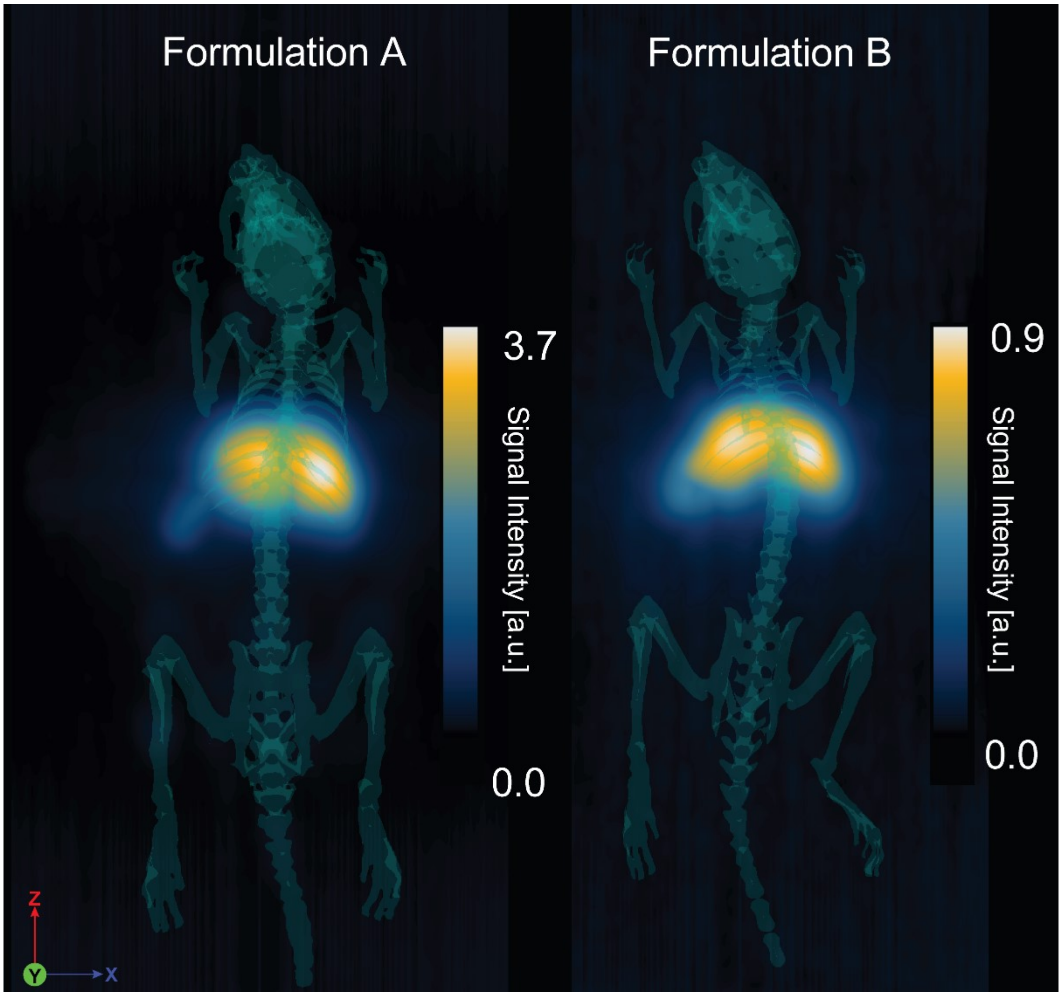

Figure S7. Skeleton outline from CT overlayed with 3D MPI Images of mice injected with formulation A (left) and formulation B (right). MPI color gradient generated from each image's maximum signal intensity to zero.
